# Antioxidant Activities, Phenolic Profiles, and Organic Acid Contents of Fruit Vinegars

**DOI:** 10.3390/antiox8040078

**Published:** 2019-03-27

**Authors:** Qing Liu, Guo-Yi Tang, Cai-Ning Zhao, Ren-You Gan, Hua-Bin Li

**Affiliations:** 1 Guangdong Provincial Key Laboratory of Food, Nutrition and Health, Department of Nutrition, School of Public Health, Sun Yat-sen University, Guangzhou 510080, China; liuq248@mail2.sysu.edu.cn (Q.L.); tanggy5@mail2.sysu.edu.cn (G.-Y.T.); zhaocn@mail2.sysu.edu.cn (C.-N.Z.); 2 Department of Food Science & Technology, School of Agriculture and Biology, Shanghai Jiao Tong University, Shanghai 200240, China

**Keywords:** fruit vinegar, antioxidant capacity, phenolics, flavonoid, organic acid

## Abstract

Fruit vinegars are popular condiments worldwide. Antioxidants and organic acids are two important components of the flavors and health benefits of fruit vinegars. This study aimed to test the antioxidant activities, phenolic profiles, and organic acid contents of 23 fruit vinegars. The results found that the 23 fruit vinegars varied in ferric-reducing antioxidant power (FRAP, 0.15–23.52 μmol Fe(II)/mL), Trolox equivalent antioxidant capacity (TEAC, 0.03–7.30 μmol Trolox/mL), total phenolic content (TPC, 29.64–3216.60 mg gallic acid equivalent/L), and total flavonoid content (TFC, 2.22–753.19 mg quercetin equivalent/L) values. Among the 23 fruit vinegars, the highest antioxidant activities were found in balsamic vinegar from Modena (Galletti), Aceto Balsamico di Modena (Monari Federzoni), red wine vinegar (Kühne), and red wine vinegar (Galletti). In addition, polyphenols and organic acids might be responsible for the antioxidant activities of fruit vinegars. The most widely detected phenolic compounds in fruit vinegars were gallic acid, protocatechuic acid, chlorogenic acid, caffeic acid, and *p*-coumaric acid, with tartaric acid, malic acid, lactic acid, citric acid, and succinic acid the most widely distributed organic acids. Overall, fruit vinegars are rich in polyphenols and organic acids and can be a good dietary source of antioxidants.

## 1. Introduction

Traditional vinegar is made from cereals and has been consumed for a long time. Another type of vinegar, fruit vinegar, made from fruit or fruit juices, has become increasingly popular in recent years because consumers are paying more attention to the functional properties of food products.

Oxidative stress is one of the main causes of certain chronic diseases such as liver, neurodegenerative, and cardiovascular diseases [1,2], which can be prevented by antioxidants [3]. Fruits are rich in antioxidants and are widely consumed by humans. Fruit vinegars can retain a number of antioxidants from fruit or fruit juices [4] and possess relatively high antioxidant capacities compared to wine and fruit juices [5]. Furthermore, fruit vinegars can increase antioxidant capacities of diets [6] as the fermentation process can produce functional components such as organic acids [7] which are not, or are only rarely, present in raw fruit materials. Fruit vinegars have also been reported to possess several health benefits, such as suppressing obesity-induced oxidative stress [8], regulating lipid metabolism, and decreasing liver damage [9], which can be at least partly due to the antioxidant activity of fruit vinegars [10]. Hence, it is valuable to determine and compare the antioxidant capacities of different fruit vinegars.

In addition, raw fruit materials are the main sources of phenolic compounds in fruit vinegars [11], and phenolics play key roles in the organoleptic properties and health effects of fruit vinegars. However, scientific research has reported that different fruit vinegars vary in their phenolic composition and contents [12], due to differences among the raw materials and manufacturing processes [7]. Only one study revealed the differences in phenolic profiles among three fruit vinegars [13]. Therefore, the phenolic profile is another important factor in measuring the value of fruit vinegars. 

Fermentation is a key process in the production of fruit vinegars, during which most organic acids are produced through chemical and microbial actions [7]. Organic acids can contribute to the organoleptic qualities of fruit vinegars [14]. Furthermore, organic acids demonstrate antimicrobial activities [15] and can control blood glucose levels and regulate lipid abnormalities [16]. The organic acids in fruit vinegars have been found to be different from those in traditional cereal vinegars [13]. Therefore, it is valuable to understand the organic acid profile of fruit vinegars.

This research, therefore, was conducted to determine the antioxidant activities, total phenolic contents (TPC), and total flavonoid contents (TFC) of 23 commonly-consumed fruit vinegars. Moreover, the main phenolic compounds and organic acids were also identified and quantified in the 23 fruit vinegars. This study provides a good reference for the public as to consuming fruit vinegars rich in antioxidant phenolics and organic acids.

## 2. Materials and Methods

### 2.1. Chemicals and Materials

The chemicals for the determination of ferric-reducing antioxidant power (FRAP), Trolox equivalent antioxidant capacity (TEAC), TPC, TFC and phenol analysis were bought according to the paper [17] we published previously. Eighteen standard phenolic compounds, including gallic acid, protocatechuic acid, gallo catechin, chlorogenic acid, cyanidin-3-glucoside, caffeic acid, epicatechin, catechin gallate, p-coumaric acid, ferulaic acid, melatonin, 2-hydroxycinnamic acid, rutin, resveratrol, daidzein, equol, quercetin, and genistein were purchased from Sigma-Aldrich (St. Louis, MO, USA). Standard organic acids, including ascorbic acid, lactic acid, citric acid, and succinic acid, were obtained from Sigma-Aldrich, and oxalic acid, tartaric acid, and malic acid were bought from National Institutes for Food and Drug Control (Beijing, China). Phosphoric acid and potassium phosphate monobasic used for organic acid analysis were of analytical grade and bought from Damao Chemical Factory (Tianjin, China) and Yongda Chemical Reagent Company (Tianjin, China), respectively. Double-distilled water was used in all the experiments. The 23 fruit vinegars (Table 1) were bought from online shopping platforms and local markets in Guangzhou, China, and were stored at 4 °C before use. 

### 2.2. Determination of FRAP, TEAC, TPC, and TFC Values

The FRAP, TEAC, TPC, and TFC values were evaluated based on the methods published previously [17], and were expressed as μmol Fe(II)/mL, μmol Trolox/mL, mg gallic acid equivalent (mg GAE)/L, and mg of quercetin equivalent (mg QE)/L, respectively.

### 2.3. Phenolic Composition Analysis

The phenolic components in 23 fruit vinegars were analyzed by High Performance Liquid Chromatography coupled with Photometric Diode Array detector (HPLC-PDA) (Waters, Milford, MA, USA) based on the literature [17]. Separation was conducted using an Agilent Zorbax Extend-C18 column (250 × 4.6 mm, 5 μm) (CA, USA) at 40 °C. Mobile phase A was formic acid solution (0.1%, *v*/*v*), and B was methanol. The procedure of gradient elution was set as: 0 min, 5% (B); 15 min, 20% (B); 20 min, 30% (B); 25 min, 37% (B); 40 min, 40% (B); 60 min, 50% (B); 65 min, 50% (B); 65.1 min, 5% (B); and 70 min, 5% (B). The spectra were scanned between 200 and 600 nm. Peak area was used to quantify phenolic compounds and the results were expressed as μg/mL.

### 2.4. Organic Acid Analysis

HPLC-PDA was used to analyze the organic acids in 23 fruit vinegars based on the literature [13] with slight modifications. Separation was conducted using an Agilent TC-C18(2) column (250 × 4.6 mm, 5 μm) at 35 °C with a mobile phase of 0.01 mol/L monopotassium phosphate buffer solution (pH = 2.5). The injection volume was 20 μL and the flow rate was 1 mL/min. The spectra were recorded at 210 nm. Peak area was used to quantify organic acids and the results were expressed as μg/mL.

### 2.5. Data Analysis

Each test was conducted in triplicate, and the results are shown as mean ± standard deviation (SD). SPSS 22.0 (IBM, Somers, NY, USA) and Excel 2007 (Redmond, WA, USA) were used to analyze the statistical differences. One-way analysis of variance (ANOVA) and the *post hoc* Tukey test were conducted to compare the differences among the means in more than two samples. The Pearson test was used for the correlation analysis. Statistical significance was defined as *p* < 0.05.

## 3. Results and Discussion

### 3.1. Antioxidant Activities of Fruit Vinegars

The antioxidant activities of phenolic compounds and other phytochemicals in natural foods are often multifunctional and more than one method is required to measure the antioxidant capacities of fruit vinegars because methods measure different aspects of antioxidant capacities [18]. In this study, FRAP and TEAC assays were both used to evaluate the antioxidant activities of fruit vinegars. The FRAP assay measures the ability to reduce a ferric tripyridyltriazine complex to the ferrous complex [19], while the TEAC method determines the ability to scavenge ABTS^•+^ free radicals [20].

The FRAP values of the 23 fruit vinegars ranged from 0.15 to 23.52 μmol Fe(II)/mL, while the TEAC values ranged from 0.03 to 7.30 μmol Trolox/mL (Table 1). These results were consistent with a previous study in which the FRAP value of a wine vinegar was 9.50 mmol Fe(II)/L, while the TEAC value was 3.12 mmol Trolox/L [5]. The five highest FRAP values, in decreasing order, were found in balsamic vinegar of Modena (Galletti) (23.52 μmol Fe(II)/mL), Aceto Balsamico di Modena (Monari Federzoni) (13.39 μmol Fe(II)/mL), red wine vinegar (Galletti) (8.04 μmol Fe(II)/mL), red wine vinegar (Kühne) (5.35 μmol Fe(II)/mL), and apple vinegar beverage (Long He Kuan) (3.99 μmol Fe(II)/mL). Similarly, results for the fruit vinegars with the highest TEAC values were generally consistent with the results for the FRAP values (Table 1).

### 3.2. TPC and TFC Values

The TPC values of fruit vinegars ranged from 29.64 to 3216.60 mg GAE/L (Table 1). The fruit vinegars with the highest TPC values were balsamic vinegar of Modena (Galletti) (3216.60 mg GAE/L), followed by Aceto Balsamico di Modena (Monari Federzoni) (1901.92 mg GAE/L), red wine vinegar (Galletti) (993.51 mg GAE/L), red wine vinegar (Kühne) (654.95 mg GAE/L), and apple vinegar (Heng Shun) (495.52 mg GAE/L). The TPC values of the tested fruit vinegars were in accordance with the findings of Ren et al. [13], in which the TPC values of fruit vinegars ranged from 274.08 to 754.50 mg GAE/L, but only three fruit vinegars were tested in that study. On the other hand, the TFC values ranged from 2.22 to 753.19 mg QE/L (Table 1). The fruit vinegars with the highest TFC values were Aceto Balsamico di Modena (Monari Federzoni) (753.19 mg QE/L), followed by balsamic vinegar of Modena (Galletti) (699.67 mg QE/L), red wine vinegar (Kühne) (51.47 mg QE/L), red wine vinegar (Galletti) (50.34 mg QE/L), and apple vinegar (Heng Shun) (31.39 mg QE/L).

Taking the FRAP, TEAC, TPC, and TFC values together, balsamic vinegar of Modena (Galletti), Aceto Balsamico di Modena (Monari Federzoni), red wine vinegar (Kühne), and red wine vinegar (Galletti) showed the highest antioxidant capacities and phenolic contents among the 23 fruit vinegars tested. In general, fruit vinegars made from red grapes, especially balsamic vinegar (balsamic vinegar of Modena (Galletti) and Aceto Balsamico di Modena (Monari Federzoni)), possessed stronger antioxidant activities. This finding is consistent with a previous study in which balsamic vinegars made from red grapes displayed significantly higher TPC values, radical scavenging, and oxidant reducing activities compared to fruit vinegars made from red grapes, white grapes, and apples [21]. In addition, antioxidant activities were found to be higher in red grape balsamic vinegars than in red wine vinegars, probably due to the phenolic contents in different fruit vinegars being affected by the raw materials, such as red grapes, white grapes and apples, and manufacturing processes [22,23]. 

### 3.3. Correlations Among FRAP, TEAC, TPC, and TFC Values

The FRAP values of the 23 fruit vinegars were highly correlated with the TEAC values (*R*^2^ = 0.989) (Table 2), indicating that the components responsible for reducing oxidants were consistent with those scavenging free radicals in fruit vinegars. In addition, a moderate correlation (*R*^2^ = 0.832) was found between the TPC and TFC values (Table 2), indicating that flavonoids were not the only phenolic compounds in fruit vinegars. In addition, the FRAP and TEAC values both showed high positive correlations with TPC values (*R*^2^ = 0.990 and 0.971, respectively) (Table 2), suggesting that phenolic components contribute to both the oxidant-reducing and radical scavenging activities of fruit vinegars. In a previous study conducted by Dávalos et al. [12], the antioxidant activities and TPC values of wine vinegars exerted a positive correlation (*p* < 0.01), consistent with our finding. On the other hand, the FRAP and TEAC values both showed moderate correlations with TFC values (*R*^2^ = 0.804 and 0.767, respectively) as shown in Table 2. Although the four values were correlated with each other, further studies are still needed to evaluate the specific compounds that contribute to each value in fruit vinegars, as most of the methods were based on the same reaction mechanism.

### 3.4. Polyphenols and Organic Acids in Fruit Vinegars

**Figure 1:**
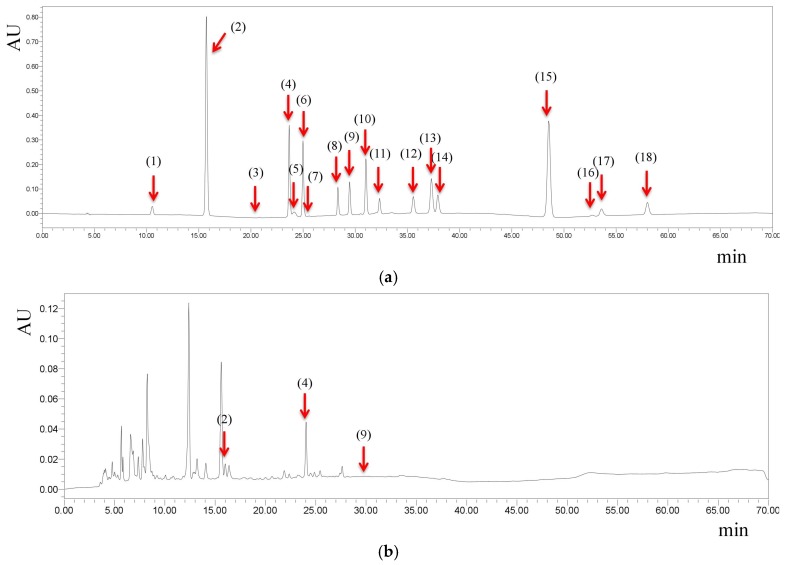
The chromatograms of phenolic compound standards (**a**) and apple vinegar (Zi Lin) (**b**) under 254 nm. Peak identification, retention time and maximum absorption: (1) gallic acid, 10.543 min, 271.3 nm; (2) protocatechuic acid, 15.723 min, 259.4 nm; (3) gallo catechin, 20.821 min, 270.1 nm; (4) chlorogenic acid, 23.661 min, 326.0 nm; (5) cyanidin-3-glucoside, 24.145 min, 279.6 nm; (6) caffeic acid, 24.987 min, 323.6 nm; (7) epicatechin, 25.580 min, 278.4 nm; (8) catechin gallate, 28.334 min, 277.2 nm; (9) *p*-coumaric acid, 29.454 min, 309.3 nm; (10) ferulaic acid, 31.018 min, 323.6 nm; (11) melatonin, 32.325 min, 221.7 nm; (12) 2-hydroxycinnamic acid, 35.562 min, 276.0 nm; (13) rutin, 37.296 min, 255.9 nm; (14) resveratrol, 37.908 min, 304.6 nm; (15) daidzein, 48.543 min, 248.8 nm; (16) equol, 52.706 min, 280.8 nm; (17) quercetin, 53.591 min, 254.7 nm; (18) genistein, 58.002 min, 259.4 nm.

Some studies have suggested that fruit vinegars possess the ability to improve oxidative stress-related disorders, such as obesity [8], liver damage [9], and diabetes [24]. Our results indicated that polyphenols in fruit vinegars were the major ingredients contributing to the antioxidant activities. We therefore further investigated the main phenolic compounds in fruit vinegars (Table 3). Retention time and UV spectra were used to recognize the phenolic compounds, and the peak areas were used to quantify these phenolic compounds (Figure 1). It was found that gallic acid, protocatechuic acid, chlorogenic acid, caffeic acid, and *p*-coumaric acid were the most widely detected phenolic compounds in 23 fruit vinegars. In addition, the highest concentrations of gallic acid, protocatechuic acid, chlorogenic acid, caffeic acid, and *p*-coumaric acid were found in Balsamic vinegar of Modena (Galletti) (12.56 μg/mL), balsamic vinegar of Modena (Galletti) (3.29 μg/mL), apple vinegar (Zi Lin) (10.91 μg/mL), balsamic vinegar of Modena (Galletti) (3.58 μg/mL), and balsamic vinegar of Modena (Galletti) (1.97 μg/mL), respectively. The data we obtained were in accordance with the data in Phenol-Explorer (a database on polyphenol content in foods), in which the contents of gallic acid, protocatechuic acid caffeic acid, and *p*-coumaric acid in vinegars were 2.59 ±, 0.81, 0.28, 0.29 mg/100 mL, respectively [25]. Balsamic vinegar of Modena (both Galletti and Monari Federzoni) contained high gallic acid and *p*-coumaric acid contents which might be responsible for the strong antioxidant capacities and high phenolic contents and needs further study. Many polyphenols, such as gallic acid, protocatechuic acid, chlorogenic acid, caffeic acid, and *p*-coumaric acid found in fruit vinegars, have been reported to suppress oxidative stress-related damages [26,27,28,29].

**Figure 2:**
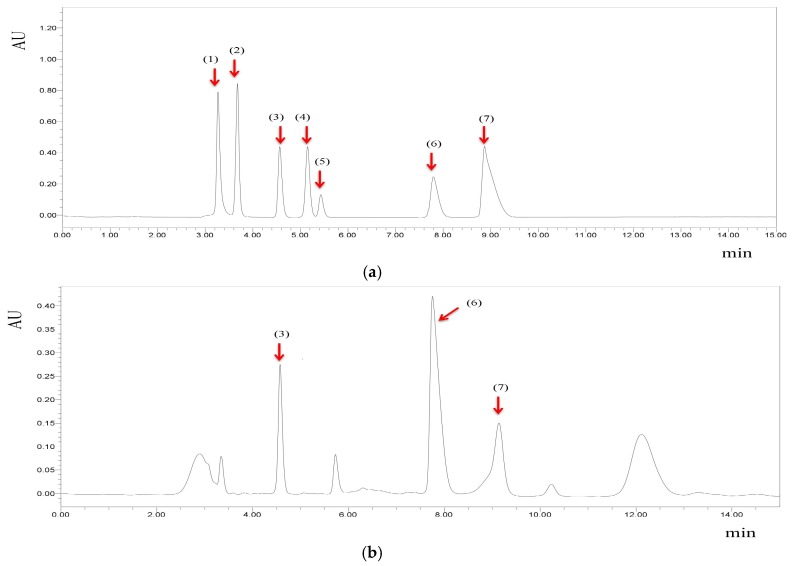
The chromatograms of the organic acid standards (**a**) and apple vinegar (Cu Bo Shi) (**b**) under 210 nm. Peak identification and retention time: (1) oxalic acid, 3.268 min; (2) tartaric acid, 3.677 min; (3) malic acid, 4.565 min; (4) ascorbic acid, 5.150 min; (5) lactic acid, 5.429 min; (6) citric acid, 7.795 min; (7) succinic acid, 8.869 min.

Organic acids are another important component of fruit vinegars. The main organic acids in fruit vinegars and their contents are shown in Table 4. Retention times were used to identify organic acids, and peak areas were used to quantify the contents (Figure 2). In the 23 fruit vinegars tested, tartaric acid, malic acid, lactic acid, citric acid, and succinic acid were the most widely detected organic acids, with the highest content found in white wine vinegar (Kühne) (1566.48 μg/mL), apple vinegar (Guang Wei Yuan) (7691.98 μg/mL), apple vinegar (Guang Wei Yuan) (2541.64 μg/mL), apple vinegar (Guang Wei Yuan) (6485.24 μg/mL), and apple vinegar (Cu Bo Shi) (1775.77 μg/mL), respectively.

Fruit vinegars are popular all over the world due to their good flavor and health benefits. Phenolic compounds and organic acids are the main components that contribute to the sensory qualities and health benefits of fruit vinegars. Some research has indicated that different grape varieties possess different phenolic contents and composition [30], and similarly apple varieties [31], depending on factors like cultivars, growing environments, and ripeness stage [17]. Factors, such as yeast strains [32], acetic acid bacteria, and production technology [33] within the fermentation processes, could also affect the phenolic profiles of fruit vinegars [4,34]. In addition, phenolic compounds have been widely explored for the abilities in preventing chronic diseases, including anti-cancer, anti-obesity, anti-aging and anti-diabetes activities [35]. According to our results, a usual serving of fruit vinegar (10 mL) contains approximately 0.30–32.67 mg GAE of polyphenols, and the FRAP and TEAC values of 10 mL fruit vinegar are approximately 1.50–235.20 μmol Fe(II) and 0.30–73.00 μmol Trolox, respectively. As the phenolic contents and composition in fruit vinegars varied, further studies are needed to explore the bioavailability and health benefits of fruit vinegars in vivo.

Organic acids in fruit vinegars are produced through hydrolysis, biochemical metabolism and microbial actions in the fermentation process. In fruit vinegars, the contents and types of organic acids affect their sensory qualities and also their health functions. Our results indicated that fruit vinegars possessed abundant organic acids with different and complex compositions. Among all the tested organic acids, tartaric acid, malic acid, lactic acid, citric acid, and succinic acid were most widely distributed in the 23 fruit vinegars tested, and the results were consistent with another study [36]. Organic acids exert some health benefits such as antimicrobial activities [15], controlling blood glucose levels, and regulating lipid abnormalities [16]. Fruit vinegars were found to contain more complex compositions of organic acids than cereal vinegars [13], indicating fruit vinegars possess a richer taste and fruit flavor compared with conventional cereal vinegars.

## 4. Conclusions

The antioxidant activities, TPC, and TFC of 23 fruit vinegars were studied. The FRAP values of the fruit vinegars were in the range of 0.15–23.52 μmol Fe(II)/mL, and the TEAC values ranged from 0.03 to 7.30 μmol Trolox/mL. The TPC and TFC values ranged from 29.64 to 3216.60 mg GAE/L and from 2.22 to 753.19 mg QE/L, respectively. Balsamic vinegar of Modena (Galletti), Aceto Balsamico di Modena (Monari Federzoni), red wine vinegar (Kühne), and red wine vinegar (Galletti) exhibited the highest antioxidant activities among 23 fruit vinegars tested. The high correlation of FRAP and TEAC with TPC values indicated that the abilities of fruit vinegars to reduce oxidants and to scavenge free radicals were mainly attributed to polyphenols. Several phenolic compounds, including, gallic acid, protocatechuic acid, chlorogenic acid, caffeic acid, and *p*-coumaric acid, and organic acids including tartaric acid, malic acid, lactic acid, citric acid, and succinic acid, were mainly detected in fruit vinegars. Some of the phenolic compounds (such as gallic acid and *p*-coumaric acid) might be responsible for the high antioxidant content and strong antioxidant activities of fruit vinegars and need to be further explored. The polyphenols and organic acids of fruit vinegars might contribute to their antioxidant activities, flavors, and health effects. Overall, fruit vinegars can be good natural sources of dietary antioxidant polyphenols and organic acids, which should be of interest to food scientists, nutritionists, the public, and food producers.

## Figures and Tables

**Table 1:** The ferric-reducing antioxidant power (FRAP), Trolox equivalent antioxidant capacity (TEAC), total phenolic contents (TPC), and total flavonoid contents (TFC) values of 23 fruit vinegars.

No.	Product	Producing Place	FRAP value (μmol Fe(II)/mL)	TEAC value (μmol Trolox/mL)	TPC value (mg GAE/L)	TFC value (mg QE/L)
1	Apple vinegar (Hai Tian)	Foshan, China	0.84 ± 0.01 ^k^	0.29 ± 0.01 ^j^	149.77 ± 3.94 ^h^	7.85 ± 0.35 ^d^
2	Apple vinegar (Ba Zhen)	Dongguan, China	1.43 ± 0.04 ^i,j^	0.43 ± 0.01 ^i,j^	249.18 ± 2.42 ^g^	20.77 ± 0.16 ^d^
3	Apple vinegar (Guang Wei Yuan)	Guangzhou, China	0.80 ± 0.02 ^k^	0.23 ± 0.01 ^j^	123.67 ± 1.51 ^h,i^	12.88 ± 0.08 ^d^
4	Apple vinegar (Heng Shun)	Zhenjiang, China	2.96 ± 0.01 ^f^	1.01 ± 0.02 ^g^	495.52 ± 20.59 ^e^	31.39 ± 0.43 ^c,d^
5	Apple vinegar (Zi Lin)	Taiyuan, China	1.83 ± 0.01 ^h^	0.69 ± 0.02 ^h^	398.17 ± 8.25 ^f^	12.78 ± 0.29 ^d^
6	Apple vinegar (Cu Bo Shi)	Xinxiang, China	0.23 ± 0.01 ^l^	0.03 ± 0.00 ^k^	66.90 ± 0.87 ^h,i^	5.86 ± 0.10 ^d^
7	Apple vinegar (Sempio)	Seoul, South Korea	0.22 ± 0.01 ^l^	0.04 ± 0.00 ^k^	43.75 ± 0.34 ^i^	2.22 ± 0.16 ^d^
8	Apple vinegar (Galletti)	Gremona, Italy	1.28 ± 0.02 ^i,j^	0.49 ± 0.02 ^i^	256.13 ± 1.86 ^g^	12.32 ± 0.16 ^d^
9	Apple vinegar (Kühne)	Hamburg, Germany	1.37 ± 0.01 ^i,j^	0.53 ± 0.02 ^i^	198.14 ± 0.33 ^g,h^	11.14 ± 0.28 ^d^
10	Apple cider vinegar (Heinz)	Pittsburgh, America	1.05 ± 0.04 ^j,k^	0.31 ± 0.01 ^j^	163.28 ± 0.15 ^h^	13.11 ± 0.14 ^d^
11	Apple cider vinegar (Xin He)	Jinan, China	2.40 ± 0.05 ^g^	0.90 ± 0.03 ^g^	426.72 ± 14.52 ^e,f^	28.04 ± 0.51 ^c,d^
12	Apple vinegar beverage (Hua Sheng Tang)	Zhongshan, China	0.19 ± 0.01 ^l^	0.06 ± 0.00 ^k^	62.19 ± 0.40 ^h,i^	5.41 ± 0.22 ^d^
13	Apple vinegar beverage (Tian Di Yi Hao)	Jiangmen, China	0.15 ± 0.00 ^l^	0.04 ± 0.00 ^k^	29.64 ± 0.16 ^i^	5.79 ± 0.14 ^d^
14	Apple vinegar beverage (Long He Kuan)	Beijing, China	3.99 ± 0.04 ^e^	1.69 ± 0.03 ^e^	469.10 ± 8.79 ^e,f^	4.52 ± 0.14 ^d^
15	Red wine vinegar (Galletti)	Gremona, Italy	8.04 ± 0.11 ^c^	3.17 ± 0.06 ^c^	993.51 ± 23.19 ^c^	50.34 ± 2.43 ^c^
16	Red wine vinegar (Kühne)	Hamburg, Germany	5.35 ± 0.09 ^d^	2.09 ± 0.03 ^d^	654.95 ± 39.52 ^d^	51.47 ± 0.99 ^c^
17	Italian red wine vinegar (Ponti)	Ghemme, Italy	3.71 ± 0.02 ^e^	1.36 ± 0.01 ^f^	396.40 ± 2.68 ^f^	19.75 ± 0.49 ^d^
18	White wine vinegar (Galletti)	Gremona, Italy	1.53 ± 0.03 ^i^	0.52 ± 0.01 ^i^	229.60 ± 2.14 ^g,h^	5.58 ± 0.30 ^d^
19	White wine vinegar (Kühne)	Hamburg, Germany	1.28 ± 0.03 ^i,j^	0.46 ± 0.02 ^i^	153.90 ± 2.13 ^h^	6.54 ± 0.16 ^d^
20	Italian white wine vinegar (Ponti)	Ghemme, Italy	1.21 ± 0.03 ^j^	0.30 ± 0.00 ^j^	117.00 ± 2.82 ^h,i^	5.09 ± 0.14 ^d^
21	Balsamic vinegar of Modena (Galletti)	Modena, Italy	23.52 ± 0.33 ^a^	7.30 ± 0.16 ^a^	3216.60 ± 132.67 ^a^	699.67 ± 24.08 ^b^
22	Aceto Balsamico di Modena (Monari Federzoni)	Bomporto, Italy	13.39 ± 0.25 ^b^	4.49 ± 0.09 ^b^	1901.92 ±16.06 ^b^	753.19 ± 36.85 ^a^
23	Fruit vinegar (Wan Jia Xiang)	Taiwan, China	0.89 ± 0.03 ^k^	0.26 ± 0.01 ^j^	194. 65 ± 0.32 ^g,h^	23.54 ± 0.99 ^c,d^

^a,b,c,d,e,f,g,h,i,j,k,l^ different letters within a parameter indicate statistical significance at *p* < 0.05.

**Table 2:** Correlation analysis among FRAP, TEAC, TPC and TFC values.

Correlation Coefficient (*R*^2^)	FRAP Value	TEAC Value	TPC Value	TFC Value
FRAP value	1	0.989 *	0.990 *	0.804 *
TEAC value	-	1	0.971 *	0.767 *
TPC value	-	-	1	0.832 *
TFC value	-	-	-	1

* indicates statistical significance at *p* < 0.01.

**Table 3:** Main phenolic compounds and their contents in 23 fruit vinegars.

No.	Products	Gallic Acid	Protocatechuic Acid	Chlorogenic Acid	Caffeic Acid	*p*-Coumaric Acid	Ferulic Acid
1	Apple vinegar (Hai Tian)	-	-	2.79 ± 0.16 ^c^	-	-	-
2	Apple vinegar (Ba Zhen)	-	-	0.32 ± 0.01 ^d^	-	-	-
3	Apple vinegar (Guang Wei Yuan)	-	-	-	-	-	-
4	Apple vinegar (Heng Shun)	-	1.54 ± 0.05 ^b^	2.99 ± 0.21 ^c^	-	-	-
5	Apple vinegar (Zi Lin)	-	0.82 ± 0.04 ^e^	10.91 ± 0.80 ^a^	-	0.17 ± 0.01 ^e^	-
6	Apple vinegar (Cu Bo Shi)	-	-	-	-	-	-
7	Apple vinegar (Sempio)	-	0.08 ± 0.00 ^h^	0.11 ± 0.00 ^d^	-	-	-
8	Apple vinegar (Galletti)	-	0.37 ± 0.01 ^g^	3.13 ± 0.11 ^c^	-	-	-
9	Apple vinegar (Kühne)	-	1.09 ± 0.04 ^d^	5.30 ± 0.29 ^b^	-	0.10 ± 0.00 ^e^	-
10	Apple cider vinegar (Heinz)	-	1.00 ± 0.06 ^d^	0.23 ± 0.00 ^d^	-	-	-
11	Apple cider vinegar (Xin He)	-	-	4.67 ± 0.21 ^b^	-	-	-
12	Apple vinegar beverage (Hua Sheng Tang)	-	-	-	-	-	-
13	Apple vinegar beverage (Tian Di Yi Hao)	-	0.19 ± 0.01 ^h^	0.59 ± 0.02 ^d^	-	-	-
14	Apple vinegar beverage (Long He Kuan)	-	-	-	-	-	-
15	Red wine vinegar (Galletti)	4.10 ± 0.18 ^d^	0.47 ± 0.04 ^f,g^	-	1.48 ± 0.10 ^c^	1.13 ± 0.05 ^c^	-
16	Red wine vinegar (Kühne)	9.99 ± 0.58 ^b^	1.38 ± 0.05 ^c^	-	-	1.39 ± 0.01 ^b^	-
17	Italian red wine vinegar (Ponti)	4.36 ± 0.33 ^d^	0.49 ± 0.01 ^f^	-	1.73 ± 0.01 ^b^	0.81 ± 0.02 ^d^	-
18	White wine vinegar (Galletti)	-	-	-	-	0.18 ± 0.00 ^e^	-
19	White wine vinegar (Kühne)	-	0.32 ± 0.01 ^g^	-	0.32 ± 0.01 ^d^	0.15 ± 0.01 ^e^	0.31 ± 0.01
20	Italian white wine vinegar (Ponti)	-	0.16 ± 0.00 ^h^	-	-	-	-
21	Balsamic vinegar of Modena (Galletti)	12.56 ± 0.86 ^a^	3.29 ± 0.05 ^a^	-	3.58 ± 0.14 ^a^	1.97 ± 0.05 ^a^	-
22	Aceto Balsamico di Modena (Monari Federzoni)	7.50 ± 0.60 ^c^	-	-	-	1.17 ± 0.06 ^c^	-
23	Fruit vinegar (Wan Jia Xiang)	-	-	-	-	-	-

^a,b,c,d,e,f,g^ Different uppercase letters within a column indicate statistical significance at *p* < 0.05.

**Table 4:** Main organic acids and their contents in 23 fruit vinegars.

No.	Products	Tartaric Acid (μg/mL)	Malic Acid (μg/mL)	Lactic Acid (μg/mL)	Citric Acid (μg/mL)	Succinic Acid (μg/mL)
1	Apple vinegar (Hai Tian)	-	480.72 ± 5.12 ^d^	-	-	-
2	Apple vinegar (Ba Zhen)	14.71 ± 0.27 ^e^	372.61 ± 7.95 ^d^	-	-	-
3	Apple vinegar (Guang Wei Yuan)	-	7691.98 ± 435.24 ^a^	2541.64 ± 107.29 ^a^	6485.24 ± 389.42 ^a^	-
4	Apple vinegar (Heng Shun)	-	1771.35 ± 117.77 ^c^	-	-	-
5	Apple vinegar (Zi Lin)	-	613.05 ± 41.96 ^d^	-	-	1637.36 ± 67.61 ^a^
6	Apple vinegar (Cu Bo Shi)	-	1707.76 ± 105.42 ^c^	-	5263.43 ± 215.07 ^b^	1775.77 ± 113.27 ^a^
7	Apple vinegar (Sempio)	-	588.03 ± 11.69 ^d^	-	-	-
8	Apple vinegar (Galletti)	-	402.11 ± 23.26 ^d^	-	-	-
9	Apple vinegar (Kühne)	-	236.38 ± 3.01 ^d^	-	-	-
10	Apple cider vinegar (Heinz)	-	-	-	-	-
11	Apple cider vinegar (Xin He)	-	4102.22 ± 253.95 ^b^	-	-	991.08 ± 13.89 ^b^
12	Apple vinegar beverage (Hua Sheng Tang)	-	573.18 ± 18.66 ^d^	-	-	-
13	Apple vinegar beverage (Tian Di Yi Hao)	-	594.91 ± 42.89 ^d^	-	-	-
14	Apple vinegar beverage (Long He Kuan)	-	4386.73 ± 326.85 ^b^	50.20 ± 1.06 ^b^	787.96 ± 24.81 ^c^	-
15	Red wine vinegar (Galletti)	383.29 ± 5.74 ^d^	-	-	-	252.60 ± 6.50 ^c^
16	Red wine vinegar (Kühne)	948.01 ± 54.86 ^b^	-	-	-	-
17	Italian red wine vinegar (Ponti)	1030.86 ± 75.33 ^b^	187.36 ± 4.47 ^d^	-	-	-
18	White wine vinegar (Galletti)	760.55 ± 8.73 ^c^	-	-	-	346.04 ± 6.56 ^c^
19	White wine vinegar (Kühne)	1566.48 ± 94.47 ^a^	-	-	-	-
20	Italian white wine vinegar (Ponti)	1066.64 ± 65.49 ^b^	-	-	-	-
21	Balsamic vinegar of Modena (Galletti)	96.15 ± 1.15 ^e^	-	-	-	-
22	Aceto Balsamico di Modena (Monari Federzoni)	393.77 ± 13.37 ^d^	603.48 ± 15.38 ^d^	-	-	-
23	Fruit vinegar (Wan Jia Xiang)	-	-	-	-	-

^a,b,c,d^ Different uppercase letters within a column indicate statistical significance at *p* < 0.05.
